# Causal relationship between circulating lipid traits and periodontitis: univariable and multivariable Mendelian randomization

**DOI:** 10.3389/fendo.2023.1214232

**Published:** 2023-07-31

**Authors:** Gaofu Hu, Chengjie Song, Yuxuan Yang, Wenhao Wang, Ao Wang, Mei Huang, Lihong Lei, Yanmin Wu

**Affiliations:** ^1^ Department of Periodontology, The Second Affiliated Hospital, College of Medicine, Zhejiang University, Hangzhou, China; ^2^ Periodontology Unit, University College London Eastman Dental Institute, London, United Kingdom

**Keywords:** lipid traits, periodontitis, Mendelian randomization, genetic correlation, causality

## Abstract

**Introduction:**

The correlation between dyslipidemia and periodontitis is revealed through epidemiological studies. However, the results are affected by several confounding factors. This study aims to elucidate the genetic causal association between circulating lipid traits and periodontitis by two-sample Mendelian randomization (MR) analysis.

**Methods:**

After the different screening processes, two cohorts of circulating lipid traits from the UK Biobank were used as exposure data, including five circulating lipid traits. The Periodontitis cohort was selected from the GeneLifestyle Interactions in Dental Endpoints (GLIDE) consortium as outcome data. In univariable MR, the inverse variance weighted (IVW) was used in conjunction with six additional analytical methods to assess causality. The Cochran Q test, I_GX_^2^ statistic, MR-PRESSO, and MR-Egger intercept were used to quantify heterogeneity and pleiotropy. The multivariable MR-IVW (MVMR-IVW) and MVMR-robust were mainly used as analytical methods in the multiple MR analyses.

**Results:**

The IVW estimates showed that genetically predicted Apolipoprotein A1 (apo A1) [odds ratio (OR)=1.158, 95% confidence interval (CI)=1.007–1.331, *P*-value=0.040] was potentially associated with the risk of periodontitis, but the statistical power of the results was low. Multivariable MR analysis did not reveal any significant causal relationship between apo A1 and periodontitis (OR=0.72, 95% CI=0.36–1.41, *P*-value=0.34). In the validation cohort, there was also no significant causal relationship between apo A1 and periodontitis (OR=1.079, 95% CI=0.903–1.290, *P*-value=0.401). Meanwhile, genetically predicted Apolipoprotein B (apo B), high-density lipoprotein cholesterol (HDL-C), low-density lipoprotein cholesterol (LDL-C), and triglyceride (TG) (all *P*-values>0.05) were not significantly associated with the risk of periodontitis causal inference.

**Conclusion:**

This MR analysis was unable to provide genetic evidence for the influence of these five circulating lipid traits on periodontitis. However, a more extensive study with a more comprehensive circulating lipid profile and periodontitis data is needed due to study limitations.

## 1 Introduction

Periodontitis is a chronic inflammatory disease caused by dysbiosis. It leads to periodontal soft tissue inflammation, continuous loss of alveolar bone, and eventually results in tooth loosening and loss (1). According to the fourth National Oral Health Survey data in China, 90% of people over 35 years old in three age groups have periodontal diseases of varying degrees, among which 30% suffer from stage III to stage IV periodontitis (2). Globally, the proportion of severe periodontitis amounts to 23.6%, which has become the sixth-largest epidemic (3). Therefore, it is essential to intervene in the risk factors of periodontitis, among which the systemic factors have been paid more and more attention. A considerable amount of epidemiological and experimental evidence has confirmed that various systemic diseases, such as cardiovascular diseases, diabetes, and respiratory diseases, play a vital role in the initiation and progression of periodontitis (4). Dyslipidemia is a condition with elevated plasma concentrations of total cholesterol (TC), low-density lipoprotein cholesterol (LDL-C), or triglyceride (TG), low plasma levels of high-density lipoprotein cholesterol (HDL-C), or a combination of these characteristics. Global data from 2008 showed that about 39% of adults (over 25 years old) had elevated levels of total lipid cholesterol, with the highest prevalence in Europe (5). More importantly, evidence suggests that patients with high lipid levels also have an increased level of systemic inflammation (6), which subsequently increases the risk of periodontitis in patients.

A recent US national cross-sectional study found that dyslipidemia had a significant direct impact on periodontitis and was associated with periodontitis through an indirect pathway of glycated hemoglobin (7). Another nationwide cohort study among women also showed that LDL-C levels were positively correlated, while HDL-C levels were negatively correlated with periodontitis risk. However, no such association was found with periodontitis risk in male subjects (8). In the case of hyperlipidemia combined with periodontitis, the probing depth (PD) and clinical attachment loss (CAL) of patients were positively correlated with the levels of TG, TC, and LDL-C (9, 10). Another cross-sectional study also showed that in patients with chronic periodontitis, the level of apolipoprotein B (apo B) in gingival crevicular fluid in the infected area was higher than that in the healthy area (11). It is well established that periodontitis and dyslipidemia share several risk factors, such as diabetes, age, obesity, alcohol consumption, smoking, etc. (12, 13). Despite the conflicting findings from different studies, there was still evidence indicating a possible association between circulating lipid traits and periodontitis. Traditional epidemiological studies, which had been subjected to such confounders and influenced by reverse causality, were found to be challenging to establish these associations as causation.

Mendelian randomization (MR) is an analysis method based on genome-wide association study (GWAS) data. It combines the Mendelian law of segregation with the instrumental variable approach (14). The former means that the alleles located on the homologous chromosomes of the parents will segregate at meiosis and can be randomly assigned to the offspring. The natural randomness of the process can mimic the randomized controlled study (RCT), similar to randomization effects (15). The latter can use single-nucleotide polymorphisms (SNPs) as instrumental variables (IVs) in the presence of unknown confounding factors and use the effect of SNPs and outcome to simulate the causal relationship between “exposure-outcome” (16). MR can overcome the limitations of conventional epidemiological studies, which is why we are using MR to look at disease correlation.

The selection of HDL-C, LDL-C, and TG as exposures in this trial was based on their widespread use in guidelines for dyslipidemia screening (17–19). LDL is efficient in transporting the majority of cholesterol in the bloodstream, whilst HDL plays a pivotal role in enabling reverse cholesterol transport from peripheral tissues to the liver. Both of these lipoproteins exert a considerable influence on plasma cholesterol levels (20). Furthermore, the inclusion of apo A1, known for its protective role against atherosclerosis and modulation of lipid metabolism, along with apo B, which encompasses all atherogenic lipoprotein particles, was also deemed necessary as additional exposures (18, 19, 21). To explore the causal relationship between five circulating lipid traits (apo A1, apo B, HDL-C, LDL-C, and TG) and the risk of periodontitis, this study conducted a two-sample univariable and multivariable MR study using data from the UK Biobank (UKB) (RRID: SCR_012815) and GeneLifestyle Interactions in Dental Endpoints (GLIDE) consortium. Each trait was evaluated independently for periodontitis risk.

## 2 Methods

### 2.1 Data sources

Genetic variation associated with circulating lipid traits was obtained from a recently published meta-analysis of genome-wide association studies (GWAS). The study assessed 35 blood and urine biomarkers in UKB based on their genetics after adjusting for statin usage, including the following five circulating lipid traits: apo A1 (N=290,198), apo B (N=317,412), HDL-C (N=291,830), LDL-C (N=318,340), and TG (N=318,674) (22). Genetic statistics of other circulating lipid traits were invoked as a validation cohort. This GWAS excluded individuals with sex mismatch or sex-chromosomal aneuploidy in the UKB. Also, it included five circulating lipid traits: apo A1 (N=115,082), apo B (N=115,082), HDL-C (N=115,082), LDL-C (N=115,082), and TG (N =115,082) (23). UKB is a large-scale biomedical cohort research database initiated and established by the UK government and long-term follow-up of participants’ health (24). Summary statistics for periodontitis were derived from the GWAS of the European Study of the GLIDE consortium, which included 17,353 periodontitis cases and 28,210 controls (25). The diagnosis of periodontitis cases is based primarily on the Centers for Disease and Control and Prevention/American Academy of Periodontology (CDC/AAP). Due to a lack of specific clinical examination data for each patient, it is impossible to get accurate staging and grading of periodontitis for patients included in this GWAS. The characteristics of the GWAS consortiums used for each variable are presented in **Table 1**.

**Table 1:** Characteristics of GWAS consortiums used for each variable.

Exposure/Outcomes	Consortium	Ethnicity	Sample Sizes
apo A1	UKB	European	290,198
apo B	UKB	European	317,412
HDL-C	UKB	European	291,830
LDL-C	UKB	European	318,340
TG	UKB	European	318,674
apo A1 (validation)	UKB	European	115,082
apo B (validation)	UKB	European	115,082
HDL-C (validation)	UKB	European	115,082
LDL-C (validation)	UKB	European	115,082
TG (validation)	UKB	European	115,082
Periodontitis	GLIDE	European	45,563

apo A1, Apolipoprotein A1; apo B, Apolipoprotein B; HDL-C, high-density lipoprotein cholesterol; LDL-C, low-density lipoprotein cholesterol; TG, triglyceride.

### 2.2 Study design

**Figure 1:**
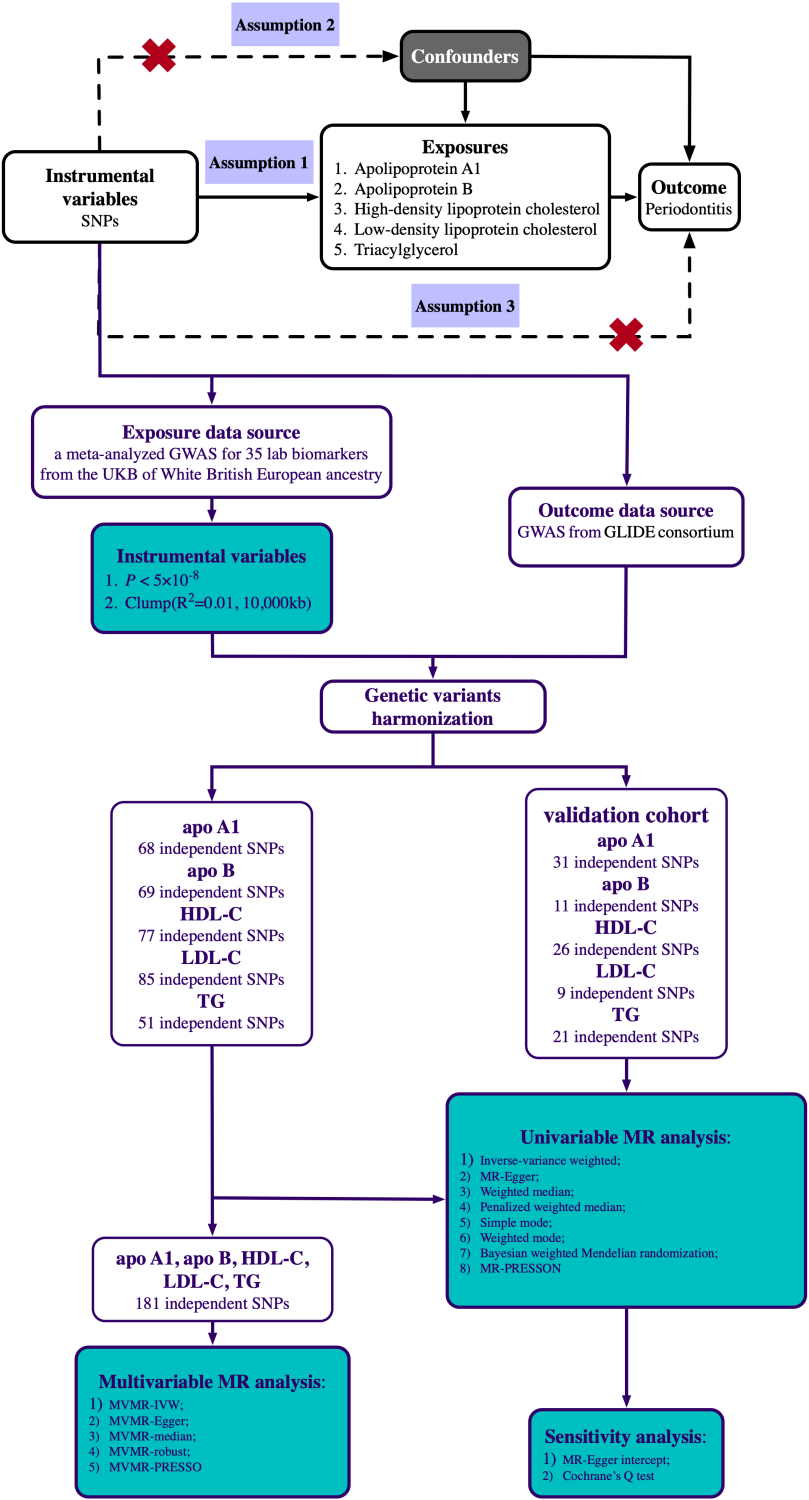
Scheme diagram of the Mendelian randomization design and flowchart of our univariable and multivariable Mendelian randomization analysis. Mendelian randomization requires valid genetic instrumental variants satisfying three assumptions. The continuous lines represent the relationships that hold in MR analysis. Dashed lines depict the association that should not be present to satisfy the second and third assumptions. SNPs, single-nucleotide polymorphisms; GWAS, genome-wide association study; UKB, UK Biobank; GLIDE, GeneLifestyle Interactions in Dental Endpoints; apo A1, Apolipoprotein A1; apo B, Apolipoprotein B; HDL-C, high-density lipoprotein cholesterol; LDL-C, low-density lipoprotein cholesterol; TG, triglyceride.

Univariable MR analysis was applied to determine if genetically predicted circulating lipid traits were significantly related to periodontitis risk, and the data from another GWAS was used for validation. Multivariable MR analysis was then conducted to determine the independent influences of the five circulating lipid traits. Genetic instrument variables (IVs) used in MR analysis to estimate the impact must comply with three main assumptions (26) (**Figure 1**): (1) Genetic variation should be significantly associated with circulating lipid traits; (2) Genetic variation must not be associated with any confounding factors; (3) Genetic variation must be associated with periodontitis only through circulating lipid traits. This study was reported following the recommendations of the latest STROBE-MR (Strengthening the Reporting of Observational studies in Epidemiology-Mendelian randomization) (**Supplementary Table 1**). Ethical approval and informed consent for this experiment can be obtained from the original study. The research design and route are shown in **Figure 1**.

### 2.3 Selection of instrumental variables

To make the IV candidates conform to the above three hypotheses, we first selected the SNPs significantly associated with apo A1, apo B, HDL-C, LDL-C, and TG in GWAS (*P*-value<5×10^-8^). Then an aggregation procedure with r^2^<0.001 and a window size=10,000 kb was performed based on 1000 Genomes Project European samples, excluding SNPs in linkage disequilibrium (LD), among which SNPs with lower *P*-values will be retained. Next, we removed SNPs that were not present in the outcome. To ensure that the selected SNPs have corresponding alleles, SNPs with inconsistent alleles and ambiguous palindromic SNPs were eliminated or adjusted. After initial screening, it was necessary to remove any potential confounding factors associated with SNPs that may affect the results. After searching for PhenoScanner V2 (http://www.phenoscanner.medschl.cam.ac.uk/, accessed on September 29, 2022) (27), blood pressure, glucose, body mass index (BMI), C-reactive protein (CRP), circulating blood cell profiles, coronary artery disease (CAD), and inflammatory bowel disease (IBD) were identified as confounding factors. To ensure the strength of the IVs, we calculated the *F*-statistic for each SNP:


$$F=\beta^{2}/SE^{2}$$

$$R^{2}=F/(N-2+F)$$

where *β* represented the genetic effect of each SNP on the exposure, *SE* was the standard error effect estimate of the corresponding GWAS, *N* was the sample size, and was the exposure variance explained by the selected IVs (28). When the *F*-statistic was much larger than 10, the IVs were less likely to be considered weak instruments (29). The determination of power in MR analyses relies on several factors, including the extent to which genetic variants account for the variance in circulating lipid traits, the overall sample size of the periodontitis GWAS, and the proportion of individuals affected by periodontitis. Subsequently, odds ratios (ORs) are computed with 80% power to address the issue of inadequate statistical capacity and guarantee the dependability of subsequent findings (30). Power calculators can be accessed at http://cnsgenomics.com/shiny/mRnd/.

### 2.4 Statistical analysis

To obtain more accurate MR estimates, we combined several two-sample MR statistical methods to assess the causal relationship between exposure and outcome. The inverse variance weighted (IVW) is the primary method for inferring causality, and it calculates the weighted average of the Wald estimates for each selected SNP (30). This method will provide the most reliable results when pleiotropy is not present in the IVs. Based on different assumptions of horizontal pleiotropy, we also employed four complementary approaches [the MR-Egger regression, the weighted median (WM), the weighted mode, and the simple mode] for inferring causality (31–33). Bayesian weighted Mendelian randomization (BWMR) further considers the uncertainty of weak effects while allowing for pleiotropy. The causal inference is performed through the variational expectation maximization (VEM) algorithm, and the results of BWMR are also reliable (34). Due to the low number of SNPs representing partial exposure after screening in the validation cohort, the penalized weighted median (PWM) method was adopted for supplementary analysis, which can minimize the impact of outliers (32). The Cochran Q test, I_GX_^2^ statistic, and MR-Egger intercept were used to quantify heterogeneity and pleiotropy (31). Second, to eliminate the influence of a single SNP, we used the leave-one-out analysis to evaluate the robustness of the results. Third, the asymmetry of the funnel plot was used to assess the reliability of the results (35). Finally, MR-PRESSO was used to test and remove horizontal pleiotropic outliers (36).

### 2.5 Multivariable Mendelian randomization analysis

Next, multivariable MR was carried out to obtain the independent effect of each circulating lipid trait on periodontitis (37). The multivariable MR analyzed the overlapping SNPs among the five circulating lipid traits after the above screening. Based on MM-estimation, using the MVMR-robust method can provide low bias and robust causality at different levels of pleiotropy (38).

For the number of exposures in this study, after the Bonferroni correction, a *P*-value<0.01 (0.05/5) was considered significantly associated, and 0.01≤*P*-value<0.05 was considered potentially associated. R statistical software, version 4.1.3 (R Foundation, Vienna, Austria) with the TwoSampleMR 0.5.6 (35), MRPRESSO 1.0 (36), BWMR 0.1.1 (34), and MVMR 0.3 (39) package was used for all analyses.

## 3 Results

### 3.1 Univariable MR analysis of the circulating lipids traits in connection with periodontitis

After the above screening process, we selected 68, 69, 77, 85, and 51 SNPs as the IVs of apo A1, apo B, HDL-C, LDL-C, and TG. The F-statistics of all SNPs were more significant than 10 (range, 29.521-1111.513), indicating that causal estimates were unlikely to be affected by weak instruments (**Supplementary Table 2**). The minimum detectable OR for each circulating lipid trait was at 80% power of periodontitis and α=5%, the expected OR were ≤0.82 or ≥1.21 for apo A1, ≤0.76 or ≥1.29 for apo B, ≤0.80 or ≥1.23 for HDL-C, ≤0.78 or ≥1.26 for LDL-C, and ≤0.69 or ≥1.40 for TG (**Supplementary Table 3**).

**Figure 2:**
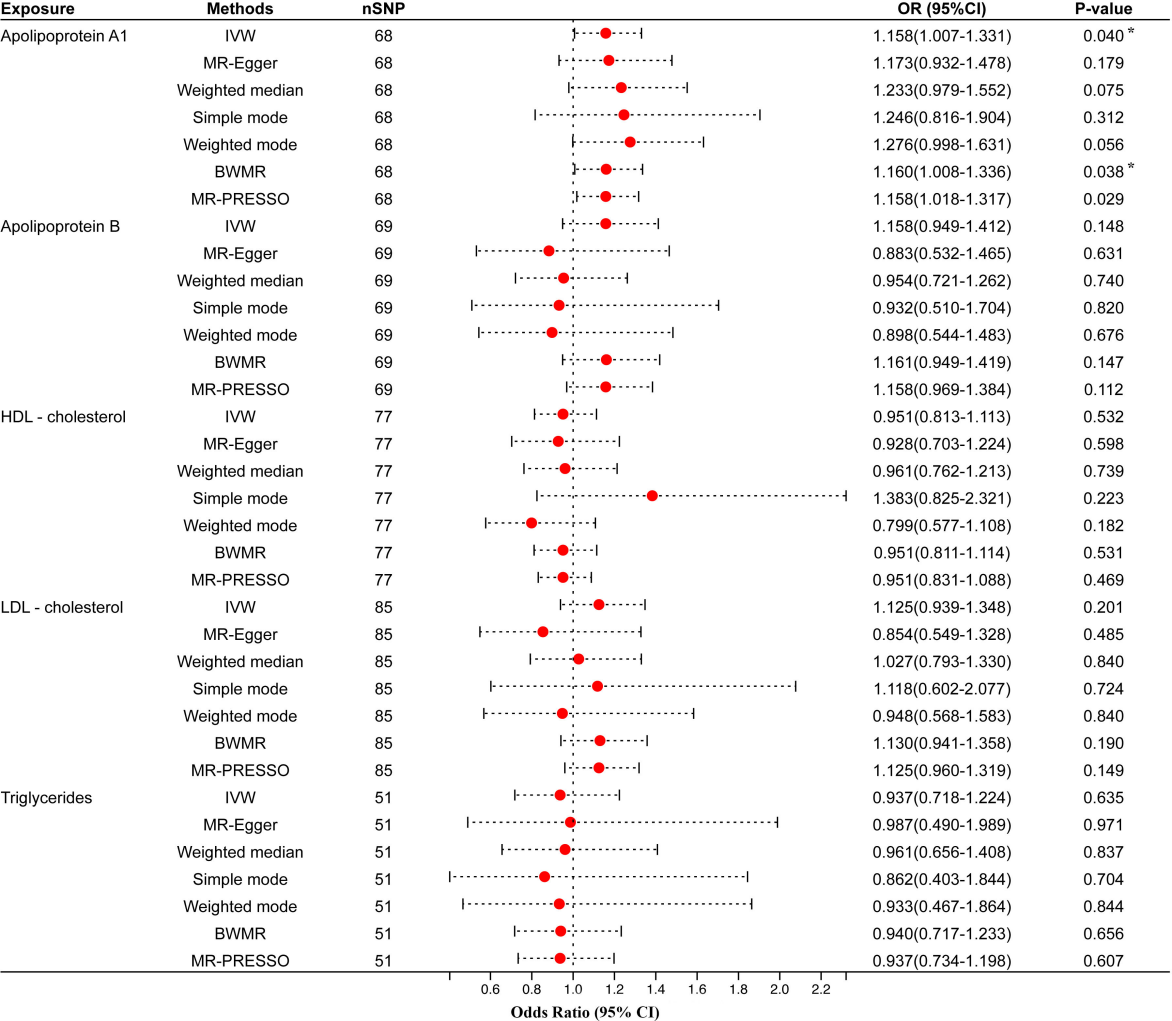
Univariable MR results of circulating lipids traits and periodontitis. The red dots represent the estimates using MR analysis, and the black bars represent the 95% confidence intervals of estimates. The OR>1 indicates increased risk while<1 indicates decreased risk. Results with *P*-value<0.05 but larger than the Bonferroni corrected significance level (0.05/5 = 0.01) were considered as suggestive associations. SNP, single-nucleotide polymorphism; OR, odds ratio; CI, confidence interval; IVW, inverse variance weighted; BWMR, Bayesian weighted Mendelian randomization; HDL- cholesterol, high-density lipoprotein cholesterol; LDL- cholesterol, low-density lipoprotein cholesterol. * Results with *P*-value<0.05 but larger than the Bonferroni corrected significance level (0.05/5=0.01) were considered as suggestive associations.

Univariable MR analysis results showed that apo A1 might have a causal connection with periodontitis, but not apo B, HDL-C, LDL-C, or TG. IVW showed that apo A1 was the only one potentially linked to periodontitis [odds ratio (OR)=1.158, 95% confidence interval (CI)=1.007–1.331, *P*-value=0.040]. Besides, the BWMR (OR=1.160, 95% CI=1.008–1.336, *P*-value=0.038) and the MR-PRESSO (OR=1.158, 95% CI=1.018–1.317, *P*-value=0.029) supported this result as well (**Figure 2**). At the same time, MR-PRESSO (*P*-value for Global Test>0.05), Cochran Q test (*P*-value>0.05), I_GX_^2^ statistics (I^2^ = 0.000), and MR-Egger test (Egger intercept=-0.0005, *P*-value>0.05) also showed that IVs had no heterogeneity and horizontal pleiotropy (**Table 2**). However, the OR calculated by the three models did not meet the minimum detectable OR of apo A1, which indicated that the causal estimation between apo A1 and periodontitis was at low strength. IVW also suggested no significant causal relationship between the remaining four circulating lipid traits and periodontitis (all *P*-values>0.05). Six other MR analyses (MR-Egger, WM, simple mode, weighted mode, BWMR, and MR-PRESSO) also showed the same results (**Figure 2**). The analysis also had no heterogeneity or horizontal pleiotropy (**Supplementary Table 4**).

**Table 2:** Heterogeneity and pleiotropy tests of circulating lipids traits causally linked to periodontitis.

Exposure	MR-PRESSO *P*-value for Global Test	IVW				MR-Egger test		
		Cochran’s Q	df	I^2^	*P*-value	Egger-intercept	SE	*P*-value
apo A1	0.794	56.795	67	0.000	0.809	-0.0005	0.004	0.888
apo B	0.875	54.853	68	0.000	0.875	0.0072	0.006	0.258
HDL-C	0.954	56.042	76	0.000	0.958	0.0009	0.004	0.832
LDL-C	0.939	64.785	84	0.000	0.941	0.0072	0.005	0.183
TG	0.759	42.249	50	0.000	0.774	-0.0012	0.008	0.877

apo A1, Apolipoprotein A1; apo B, Apolipoprotein B; HDL-C, high-density lipoprotein cholesterol; LDL-C, low-density lipoprotein cholesterol; TG, triglyceride; IVW, inverse variance weighted.

**Figure 3:**
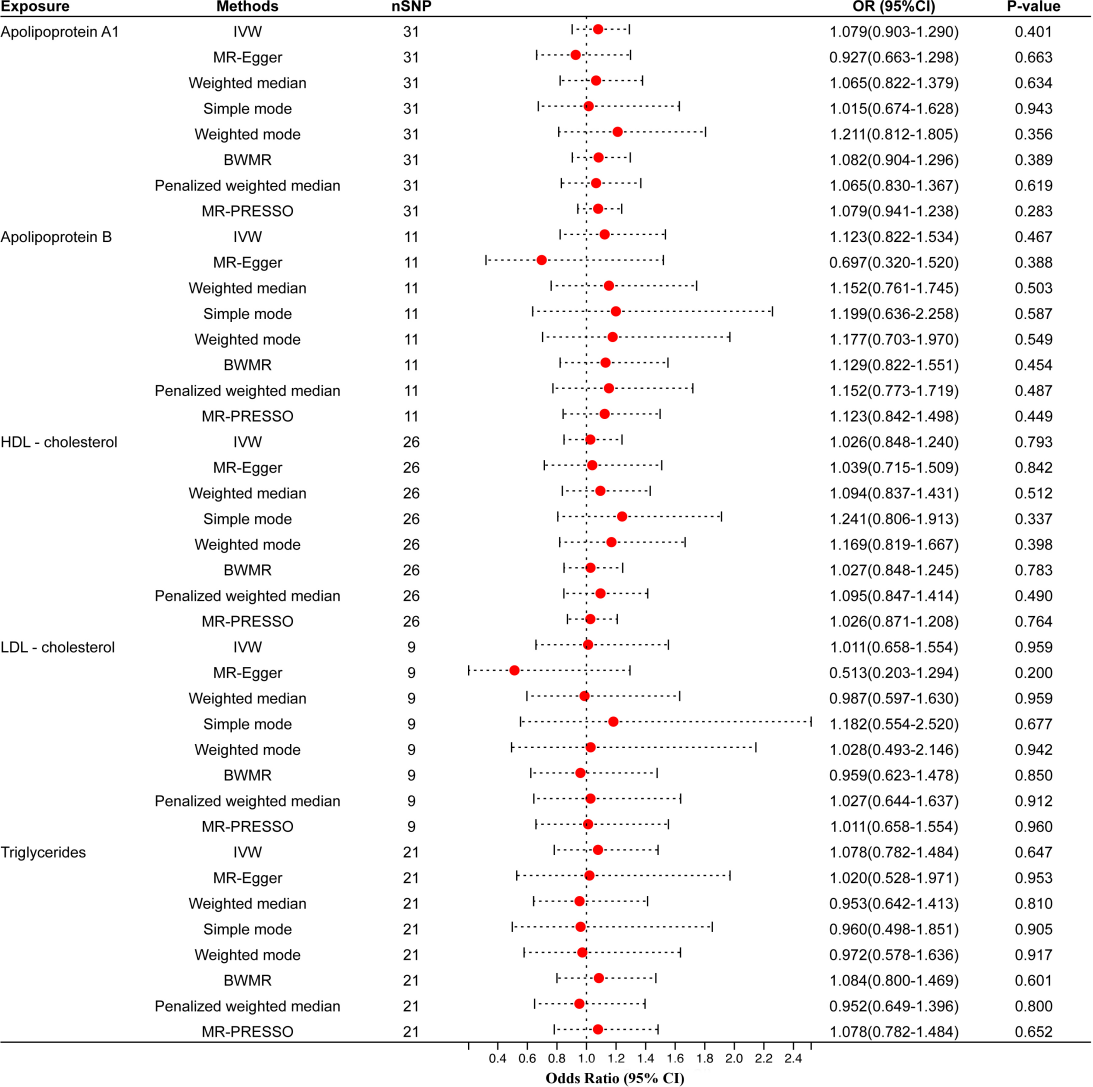
Univariable MR results of circulating lipids traits and periodontitis of the validation cohort. The red dots represent the estimates using MR analysis, and the black bars represent the 95% confidence intervals of estimates. The OR>1 indicates increased risk while<1 indicates decreased risk. SNP, single-nucleotide polymorphism; OR, odds ratio; CI, confidence interval; IVW, inverse variance weighted; BWMR, Bayesian weighted Mendelian randomization; HDL- cholesterol, high-density lipoprotein cholesterol; LDL- cholesterol, low-density lipoprotein cholesterol.

We also validated results using different GWAS on circulating lipid traits, and the univariable MR showed that none of the five circulating lipid traits had a significant causal relationship with periodontitis (**Figure 3**). The *F*-statistics of all SNPs were more extensive than 10 (range, 30.199-367.167), indicating that causal estimates were unlikely to be affected by weak instruments (**Supplementary Table 7**). There was likewise no heterogeneity or horizontal pleiotropy among all SNPs (**Table 3**).

**Table 3:** Heterogeneity and pleiotropy tests of circulating lipids traits causally linked to periodontitis in the validation cohort.

Exposure	MR-PRESSO *P*-value for Global Test	IVW				MR-Egger test		
		Cochran’s Q	df	I^2^	*P*-value	Egger-intercept	SE	*P*-value
apo A1	0.953	17.688	30	0.000	0.963	0.0077	0.007	0.304
apo B	0.621	8.542	10	0.000	0.576	0.0211	0.016	0.224
HDL-C	0.830	18.587	25	0.000	0.816	-0.0007	0.008	0.937
LDL-C	0.173	11.999	8	0.333	0.151	0.0308	0.019	0.156
TG	0.077	30.168	20	0.337	0.067	0.0026	0.014	0.853

apo A1, Apolipoprotein A1; apo B, Apolipoprotein B; HDL-C, high-density lipoprotein cholesterol; LDL-C, low-density lipoprotein cholesterol; TG, triglyceride; IVW, inverse variance weighted.

The MR-PRESSO outlier test and the leave-one-out analysis showed that all the above results were unaffected by outliers. Leave-one-out analysis diagrams, scatter plots, and funnel plots are all shown in **Supplementary Figures 1**-**10**.

### 3.2 Multivariable MR analysis of the circulating lipids traits in connection with periodontitis

**Figure 4:**
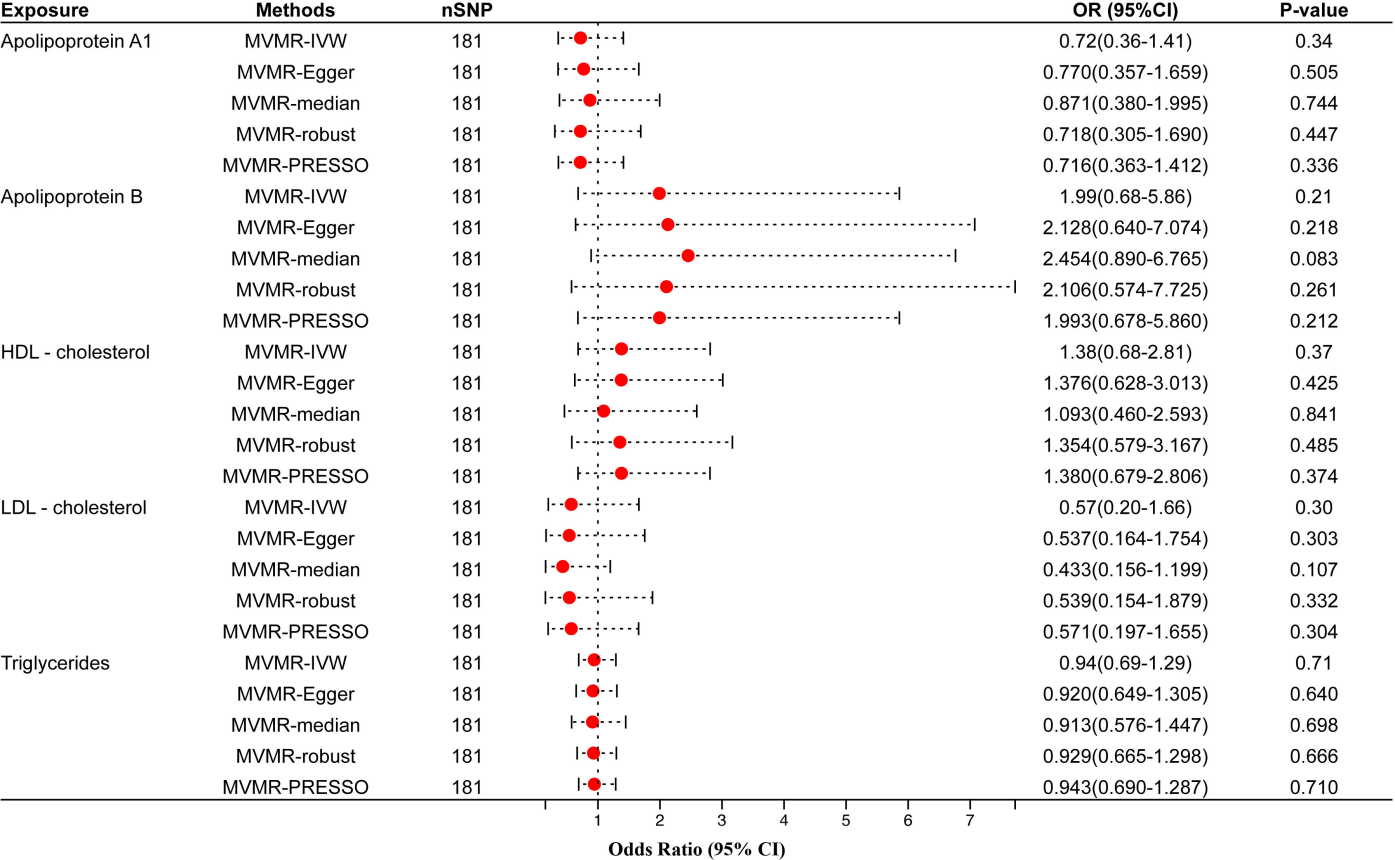
Multivariable MR results of circulating lipids traits and periodontitis. The red dots represent the estimates using MR analysis, and the black bars represent the 95% confidence intervals of estimates. The OR>1 indicates increased risk while<1 indicates decreased risk. SNP, single-nucleotide polymorphism; OR, odds ratio; CI, confidence interval; IVW, inverse variance weighted; HDL- cholesterol, high-density lipoprotein cholesterol; LDL- cholesterol, low-density lipoprotein cholesterol.

We screened 181 SNPs as IVs of multivariable MR to analyze how the different circulating lipid traits affect periodontitis independently (**Supplementary Table 8**). MVMR-IVW estimate showed that apo A1 (OR=0.72, 95% CI=0.36–1.41, *P*-value=0.34), apo B (OR=1.99, 95% CI=0.68–5.86, *P*-value=0.21), HDL-C (OR=1.38, 95% CI=0.68–2.81, *P*-value=0.37), LDL-C(OR=0.57, 95% CI=0.20–1.66, *P*-value=0.30) and TG (OR=0.94, 95% CI=0.69–1.29, *P*-value=0.71) were not significantly associated with the risk of periodontitis (**Figure 4**). The remaining four multivariable MR analysis methods also did not obtain evidence of causality. These results were consistent with the univariable MR analysis.

## 4 Discussion

This is the first study investigating the causal correlation between five circulating lipid traits and periodontitis by univariable and multivariable MR. Although univariable MR suggested a potential association of apo A1 with periodontitis, the strength of this finding was low, and analyses of the validation cohort also refuted this association. Therefore, our two-sample MR analysis did not observe robust evidence supporting a causal association of these circulating lipid traits with periodontitis.

However, epidemiological studies have pointed out that apo A1, apo B, HDL-C, LDL-C, and TG are associated with periodontitis. A cross-sectional study involving 123 patients with hyperlipidemia showed that hyperlipidemia significantly increased the severity of periodontitis (OR=2.48, 95% CI=0.63–9.76, *P*-value=0.019) (40). Another case-control study of 13,584 participants, all from the Fifth Korean National Health and Nutrition Examination Survey, showed that the TG/HDL-C ratio was a significant positive correlation with the prevalence of periodontitis after adjusting for variables such as sex, age, BMI, and dyslipidemia (OR=1.23, 95% CI=1.02–1.48, *P*-value<0.001) (41). Several cross-sectional studies also found that the mean values of salivary oxidative stress markers and periodontal clinical parameters (PD, CAL) had an extremely significant positive correlation with TG, LDL-C, and TC serum levels and were significantly negatively correlated with serum HDL-C levels (42, 43).

It is worth noting that the conclusions of observational studies can only reflect correlation and cannot draw causal inferences. We believe that the reasons for the association between the two in previous trials may be as follows: On the one hand, considering the ethical issues in randomized clinical trials on patients with hyperlipidemia, most clinical research results are from cross-sectional studies, which makes the conclusions vulnerable to reverse causality. For example, in a case-control study of 65,078 Korean participants, after a median follow-up of 5.19 years, periodontitis was associated with lower HDL-C levels (β=-0.0066 mmol/L, SE=0.0026, *P*-value = 0.013) and higher TG levels (β=0.0307 mmol/L, SE=0.0049, *P*-value< 0.001) (44). This seems to be because, during periodontitis, inflammatory factors [C-reactive protein (CRP), tumor necrosis factor-α (TNF-α), interleukin-1β (IL-1β), and IL-6] released into serum can promote adipose tissue lipolysis and inhibit lipoprotein lipase activity, increase the synthesis of TG, and reduce the clearance of TG and LDL-C (45).

On the other hand, it is difficult for existing epidemiological studies to adequately adjust for confounding factors such as gut microbiome, personal dietary habits, and lifestyle. The gut microbiome affects lipid metabolism and the development and progression of periodontitis. After performing fecal microbiota transplantation to high-fat mice, Zhou D et al. observed that the number of beneficial bacteria *Christensenellaceae* and *Lactobacillus* in the intestine increased, the disordered intestinal flora was partially corrected, lipid metabolism was also improved, and the levels of TG and total cholesterol decreased (46). Meta-analysis results also showed that the intake of probiotic preparations could increase the level of HDL-C and effectively improve dyslipidemia (47). These all indicate that the balance of intestinal flora can regulate the lipid metabolism process. Similarly, several clinical studies have shown that adjuvant probiotic *Lactobacillus reuteri* therapy can significantly improve clinical periodontal symptoms after initial periodontal treatment (48). It has also been found in animal experiments that oral administration of probiotics before and after the formation of periodontitis in mice can significantly reduce the inflammatory factors in periodontitis and relieve alveolar bone resorption and periodontal ligament destruction (49, 50). The above studies suggest that the intestinal flora may affect the immune regulation of periodontal tissue and alveolar bone metabolism through the intestinal immune system. A newly published MR study on gut microbiota and periodontitis also confirmed this causal relationship (51). Thirdly, smoking, as the common risk factor for dyslipidemia and periodontitis, would also affect clinical trials as a confounding factor (52, 53).

At the same time, other circulating lipid traits release several proinflammatory cytokines that may be involved in the initiation and progression of periodontitis when dyslipidemia occurs. Continuously aggregated lipids will undergo glycation and oxidative modifications, inducing inflammatory response and oxidative stress (54). Of those, it was confirmed that palmitic acid (PA) can activate Toll-like receptor 4 (TLR4)/the myeloid differentiation factor 8 (MyD88)/the phosphatidylinositol 3-kinase (PI3K)/protein kinase B (PKB)/the nuclear factor-κB (NF-κB) pathway and then induce the production of proinflammatory cytokines including IL-1β and IL-6 (55). Cholesterol crystals phagocytized by macrophages can also activate the NOD−like receptor protein 3 (NLRP3) inflammasome by inducing the release of cathepsin B, leading to the production and secretion of IL-1β and IL-6 (56, 57). The systemic inflammatory burden will be due to these proinflammatory cytokines through blood circulation, which may participate in the immune regulation of the periodontal microenvironment (58). On the other hand, oxidized low-density lipoprotein (ox-LDL) is induced to increase and accumulate locally due to dyslipidemia (59). Suzuki et al. observed that ox-LDL could act on human gingival epithelial cells to activate the NF-κB pathway and ultimately induce the production of IL-8 (60, 61). Some cross-sectional studies also found that as the severity of periodontitis increased, the levels of ox-LDL in patients’ serum and gingival crevicular fluid both increased (11, 40). Therefore, we speculate that dyslipidemia can directly promote the occurrence and development of periodontal inflammation by increasing the accumulation of ox-LDL in the gingival crevicular fluid.

The strength of our study is that it is the first to utilize a multivariable MR design to investigate the causal effect of serum levels of five circulating lipid traits on periodontitis risk. This is an excellent solution to the vertical pleiotropy caused by the interaction between the shape of circulating blood lipids to obtain the independent causal effect of each parameter on periodontitis. More importantly, the sample size in the two-sample MR study is vast, and its hierarchy of evidence is higher than that of observational studies, second only to RCT studies (62). At the same time, this study also has certain limitations. First, exposure and outcome data were from European populations, and the results were not broadly generalizable to other ethnicities. Second, only five circulating lipid traits were reflected in this study. Other lipid-related characteristics were not analyzed, such as very low-density lipoprotein cholesterol, chylomicrons, ox-LDL, and fatty acids. Third, the periodontitis cohort that was the result of this study did not use the latest diagnostic criteria of the European Federation of Periodontology/the American Academy of Periodontology (EFP/AAP) for periodontitis. To further explore whether each circulating blood lipid trait has different causal effects on periodontitis with dissimilar severities, it is still necessary to use GWAS with more accurate periodontitis staging and grading in the future.

## 5 Conclusion

In conclusion, our findings suggest that none of the five circulating lipid traits (apo A1, apo B, HDL-C, LDL-C, and TG) is causally associated with periodontitis. Further studies using GWAS datasets, which include more comprehensive circulating lipid traits and more accurate definitions of periodontitis, are needed to validate the findings.

## Data availability statement

The original contributions presented in the study are included in the article/**Supplementary Material**. Further inquiries can be directed to the corresponding author.

## Ethics statement

Ethical review and approval was not required for the study on human participants in accordance with the local legislation and institutional requirements. Written informed consent for participation was not required for this study in accordance with the national legislation and the institutional requirements.

## Author contributions

GH performed the univariable and multivariable MR analysis and drafted and critically revised the manuscript. CS, YY, WW, and AW contributed to the results and discussion and critically revised the manuscript. MH and LL contributed to the design of the project and critically revised the manuscript. YW conceived the study, supervised the statistical analysis, and critically revised the manuscript. All authors gave their final approval and agree to be accountable for all aspects of the work.
