# Supplementary material for: Causal relationship between circulating lipid traits and periodontitis: univariable and multivariable Mendelian randomization
Source: Front Endocrinol (Lausanne). 2023 Jul 31;14:1214232. doi: 10.3389/fendo.2023.1214232 (PMC10424932; doi:10.3389/fendo.2023.1214232)
Supplement: Supplementary file 1 [file Image_1.pdf]

Supplementary Material

Causal relationship between circulating lipid traits and periodontitis: Univariable and multivariable Mendelian randomization

Gaofu Hu1, Chengjie Song1, Yuxuan Yang1, Wenhao Wang1, Ao Wang1, Mei Huang2, Lihong Lei1, Yanmin Wu1*

*** Correspondence:** Yanmin Wu: wuyanmin@zju.edu.cn

1 Supplementary Figures and Tables

1.1 Supplementary Figures

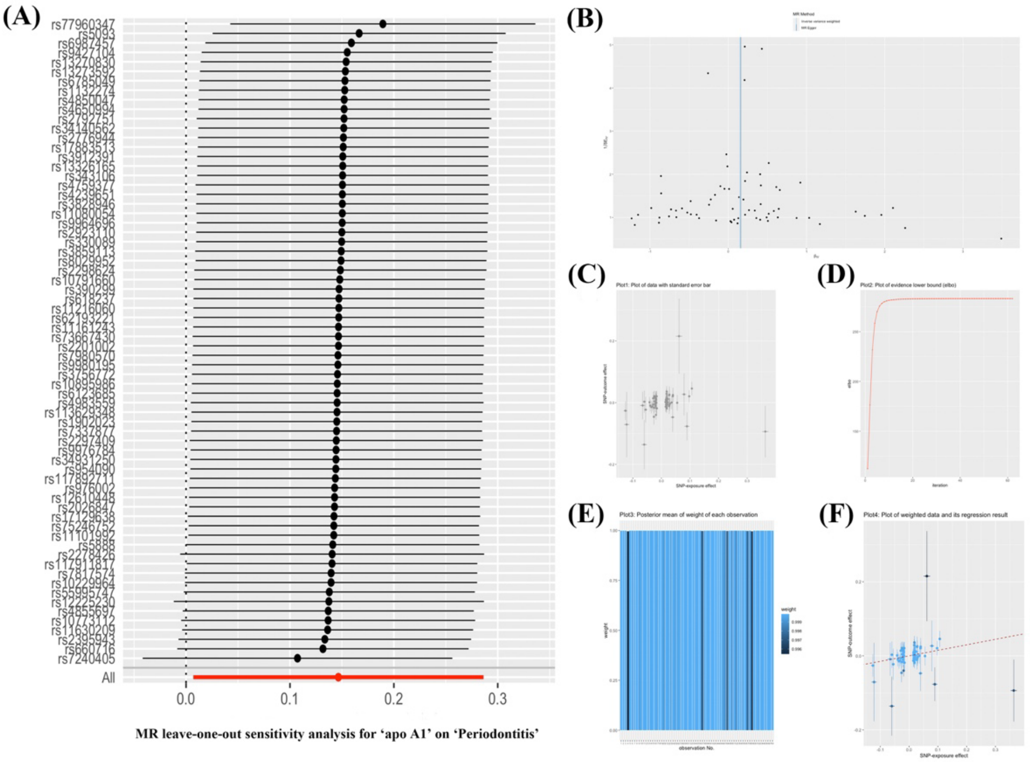

Supplementary Figure 1. Complementary MR analyses of apo A1 with periodontitis.

A. Leave-one-out sensitivity analysis, removed each SNP in turn and then performed MR repeatedly to eliminate the influence of a single SNP; B. Funnel plot, tested for potential asymmetry; C. Plot of data with standard error bar in BWMR. Dots represent the estimated causal effect sizes (Beta), and their standard errors (SE) are represented by bars; D. Plot of evidence lower in BWMR; E. Posterior means of the weight of each observation in BWMR, valid SNPs were assigned close to 1 and outliers

were adaptively down-weighted by BWMR; F. Plot of weighted data and its regression result in BWMR. The dots represent the causal effect (Beta) for each SNP; the bars represent their standard errors (SE); the dashed line indicates the regression slope for BWMR, and the depth of blue means the weight.

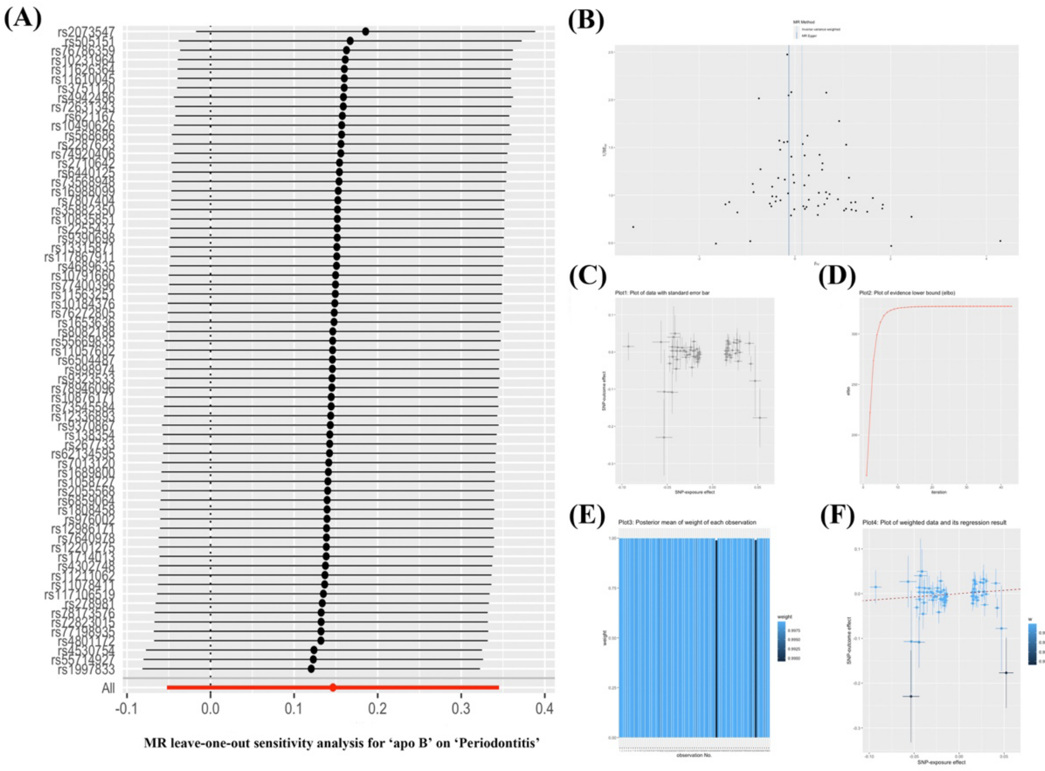

Supplementary Figure 2. Complementary MR analyses of apo B with periodontitis.

A. Leave-one-out sensitivity analysis, removed each SNP in turn and then performed MR repeatedly to eliminate the influence of a single SNP; B. Funnel plot, tested for potential asymmetry; C. Plot of data with standard error bar in BWMR. Dots represent the estimated causal effect sizes (Beta), and their standard errors (SE) are represented by bars; D. Plot of evidence lower in BWMR; E. Posterior means of the weight of each observation in BWMR, valid SNPs were assigned close to 1 and outliers were adaptively down-weighted by BWMR; F. Plot of weighted data and its regression result in BWMR. The dots represent the causal effect (Beta) for each SNP; the bars represent their standard errors (SE); the dashed line indicates the regression slope for BWMR, and the depth of blue means the weight.

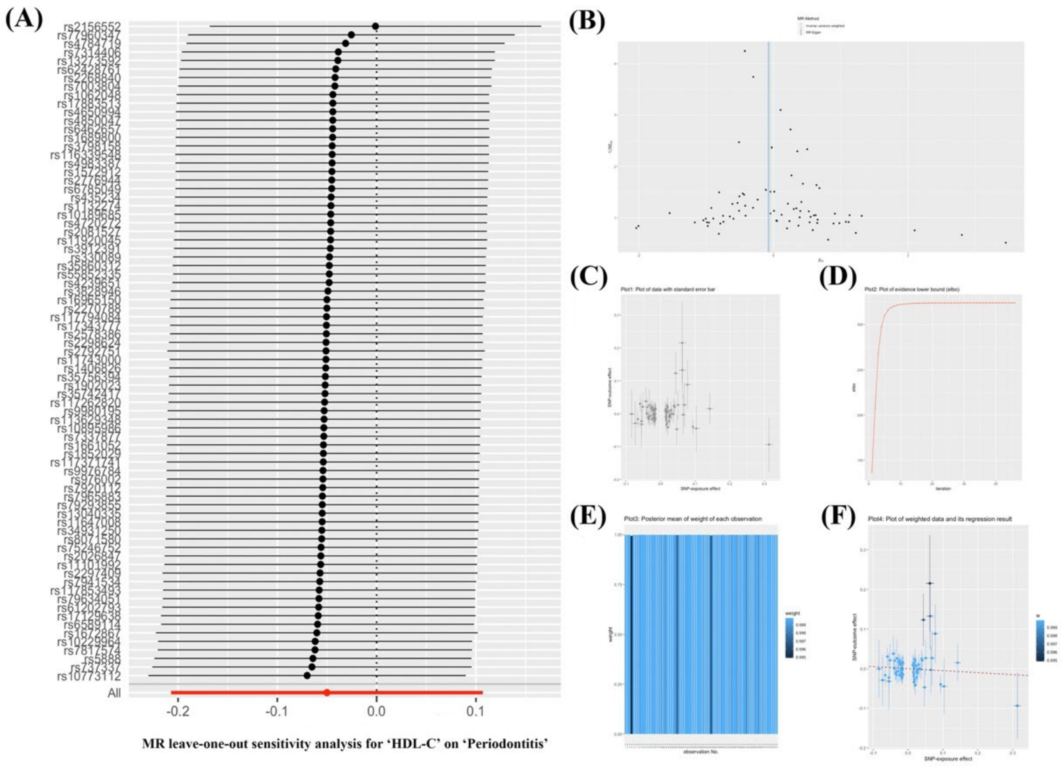

Supplementary Figure 3. Complementary MR analyses of HDL-C with periodontitis.

A. Leave-one-out sensitivity analysis, removed each SNP in turn and then performed MR repeatedly to eliminate the influence of a single SNP; B. Funnel plot, tested for potential asymmetry; C. Plot of data with standard error bar in BWMR. Dots represent the estimated causal effect sizes (Beta), and their standard errors (SE) are represented by bars; D. Plot of evidence lower in BWMR; E. Posterior means of the weight of each observation in BWMR, valid SNPs were assigned close to 1 and outliers were adaptively down-weighted by BWMR; F. Plot of weighted data and its regression result in BWMR. The dots represent the causal effect (Beta) for each SNP; the bars represent their standard errors (SE); the dashed line indicates the regression slope for BWMR, and the depth of blue means the weight.

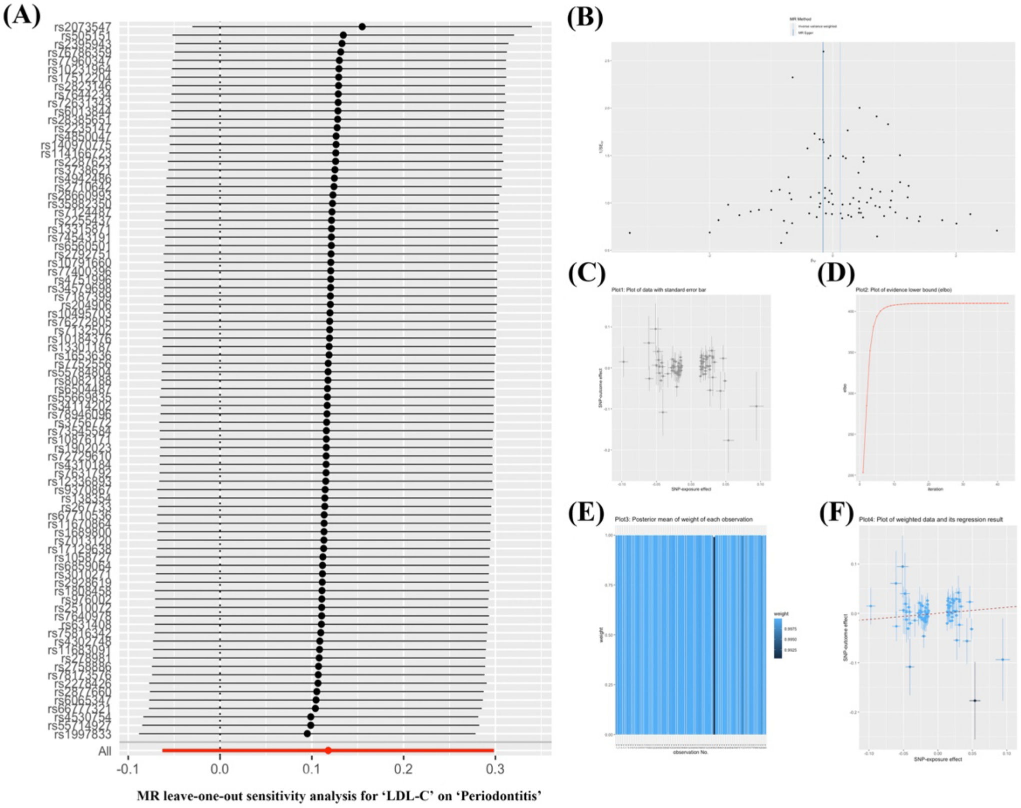

Supplementary Figure 4. Complementary MR analyses of LDL-C with periodontitis.

A. Leave-one-out sensitivity analysis, removed each SNP in turn and then performed MR repeatedly to eliminate the influence of a single SNP; B. Funnel plot, tested for potential asymmetry; C. Plot of data with standard error bar in BWMR. Dots represent the estimated causal effect sizes (Beta), and their standard errors (SE) are represented by bars; D. Plot of evidence lower in BWMR; E. Posterior means of the weight of each observation in BWMR, valid SNPs were assigned close to 1 and outliers were adaptively down-weighted by BWMR; F. Plot of weighted data and its regression result in BWMR. The dots represent the causal effect (Beta) for each SNP; the bars represent their standard errors (SE); the dashed line indicates the regression slope for BWMR, and the depth of blue means the weight.

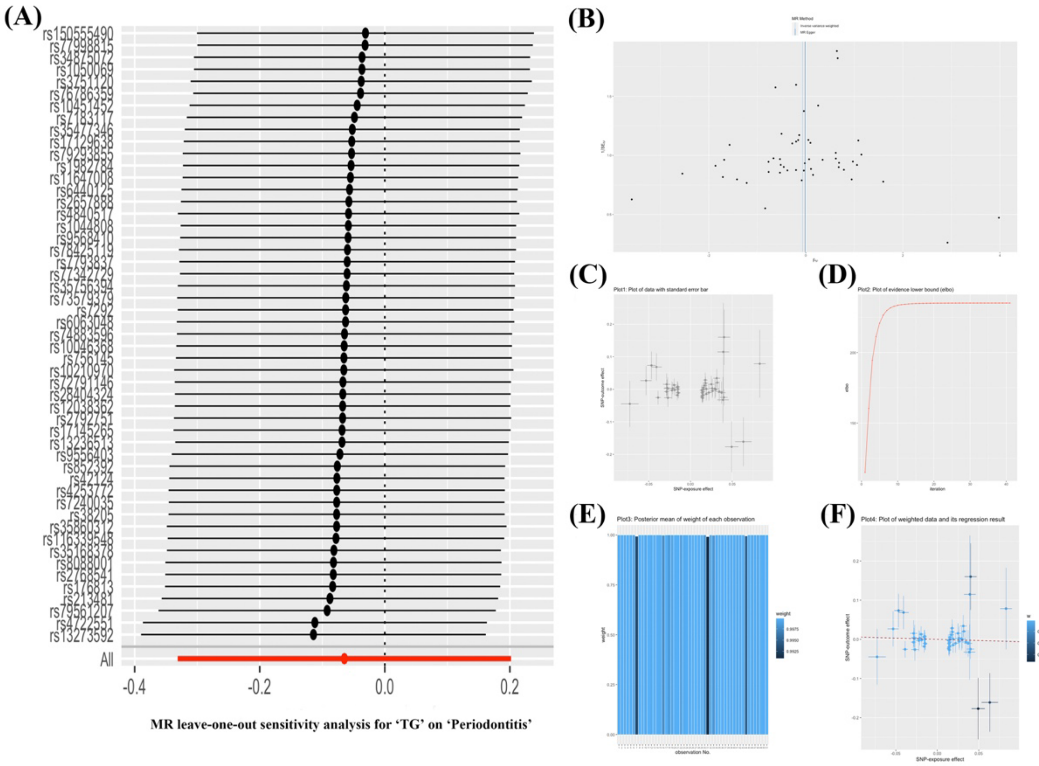

Supplementary Figure 5. Complementary MR analyses of TG with periodontitis.

A. Leave-one-out sensitivity analysis, removed each SNP in turn and then performed MR repeatedly to eliminate the influence of a single SNP; B. Funnel plot, tested for potential asymmetry; C. Plot of data with standard error bar in BWMR. Dots represent the estimated causal effect sizes (Beta), and their standard errors (SE) are represented by bars; D. Plot of evidence lower in BWMR; E. Posterior means of the weight of each observation in BWMR, valid SNPs were assigned close to 1 and outliers were adaptively down-weighted by BWMR; F. Plot of weighted data and its regression result in BWMR. The dots represent the causal effect (Beta) for each SNP; the bars represent their standard errors (SE); the dashed line indicates the regression slope for BWMR, and the depth of blue means the weight.

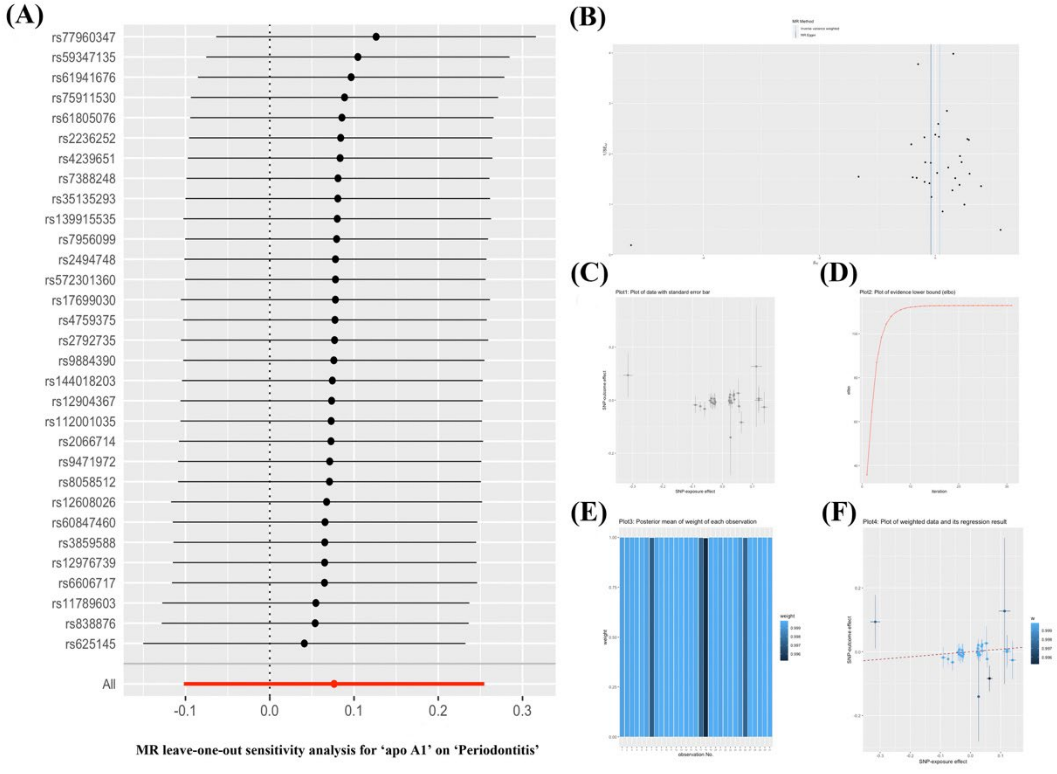

Supplementary Figure 6. Complementary MR analyses of apo A1 with periodontitis of the validation cohort.

A. Leave-one-out sensitivity analysis, removed each SNP in turn and then performed MR repeatedly to eliminate the influence of a single SNP; B. Funnel plot, tested for potential asymmetry; C. Plot of data with standard error bar in BWMR. Dots represent the estimated causal effect sizes (Beta), and their standard errors (SE) are represented by bars; D. Plot of evidence lower in BWMR; E. Posterior means of the weight of each observation in BWMR, valid SNPs were assigned close to 1 and outliers were adaptively down-weighted by BWMR; F. Plot of weighted data and its regression result in BWMR. The dots represent the causal effect (Beta) for each SNP; the bars represent their standard errors (SE); the dashed line indicates the regression slope for BWMR, and the depth of blue means the weight.

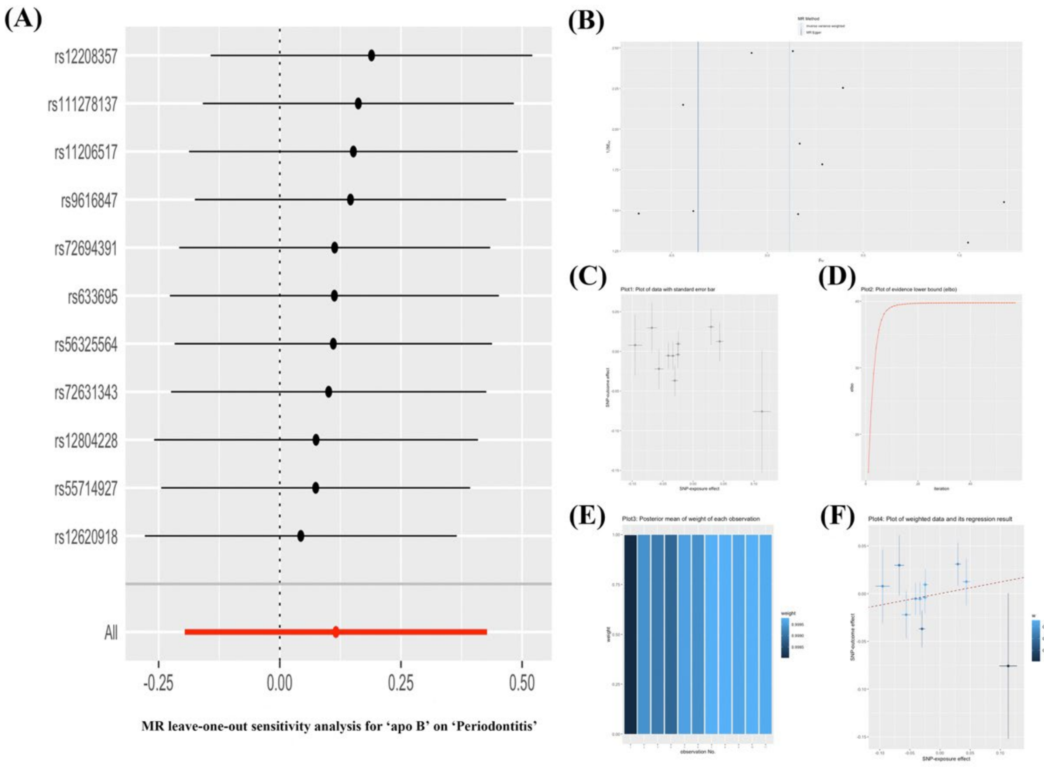

Supplementary Figure 7. Complementary MR analyses of apo B with periodontitis of the validation cohort.

A. Leave-one-out sensitivity analysis, removed each SNP in turn and then performed MR repeatedly to eliminate the influence of a single SNP; B. Funnel plot, tested for potential asymmetry; C. Plot of data with standard error bar in BWMR. Dots represent the estimated causal effect sizes (Beta), and their standard errors (SE) are represented by bars; D. Plot of evidence lower in BWMR; E. Posterior means of the weight of each observation in BWMR, valid SNPs were assigned close to 1 and outliers were adaptively down-weighted by BWMR; F. Plot of weighted data and its regression result in BWMR. The dots represent the causal effect (Beta) for each SNP; the bars represent their standard errors (SE); the dashed line indicates the regression slope for BWMR, and the depth of blue means the weight.

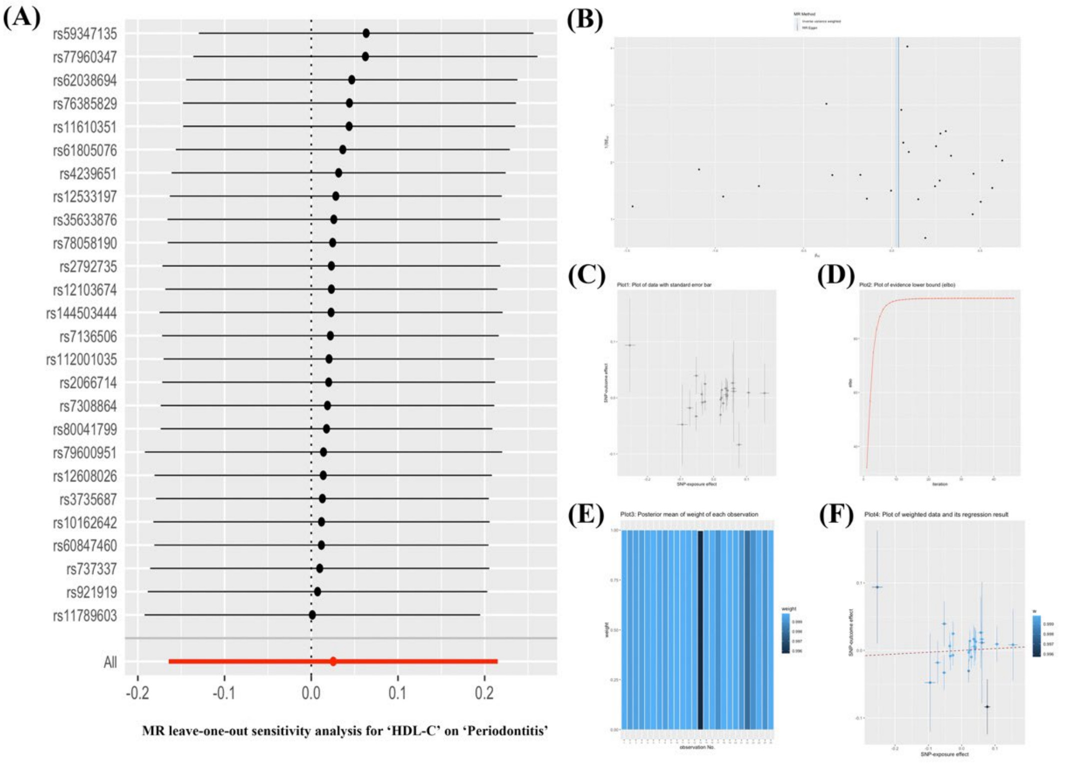

Supplementary Figure 8. Complementary MR analyses of HDL-C with periodontitis of the validation cohort.

A. Leave-one-out sensitivity analysis, removed each SNP in turn and then performed MR repeatedly to eliminate the influence of a single SNP; B. Funnel plot, tested for potential asymmetry; C. Plot of data with standard error bar in BWMR. Dots represent the estimated causal effect sizes (Beta), and their standard errors (SE) are represented by bars; D. Plot of evidence lower in BWMR; E. Posterior means of the weight of each observation in BWMR, valid SNPs were assigned close to 1 and outliers were adaptively down-weighted by BWMR; F. Plot of weighted data and its regression result in BWMR. The dots represent the causal effect (Beta) for each SNP; the bars represent their standard errors (SE); the dashed line indicates the regression slope for BWMR, and the depth of blue means the weight.

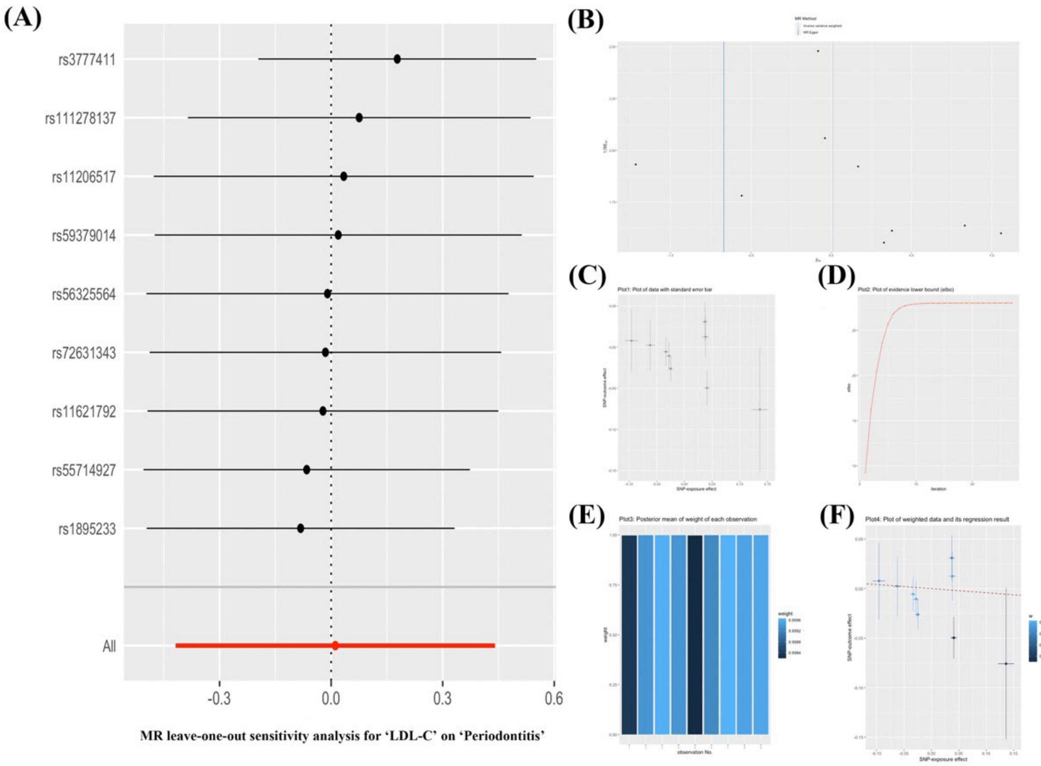

Supplementary Figure 9. Complementary MR analyses of LDL-C with periodontitis of the validation cohort.

A. Leave-one-out sensitivity analysis, removed each SNP in turn and then performed MR repeatedly to eliminate the influence of a single SNP; B. Funnel plot, tested for potential asymmetry; C. Plot of data with standard error bar in BWMR. Dots represent the estimated causal effect sizes (Beta), and their standard errors (SE) are represented by bars; D. Plot of evidence lower in BWMR; E. Posterior means of the weight of each observation in BWMR, valid SNPs were assigned close to 1 and outliers were adaptively down-weighted by BWMR; F. Plot of weighted data and its regression result in BWMR. The dots represent the causal effect (Beta) for each SNP; the bars represent their standard errors (SE); the dashed line indicates the regression slope for BWMR, and the depth of blue means the weight.

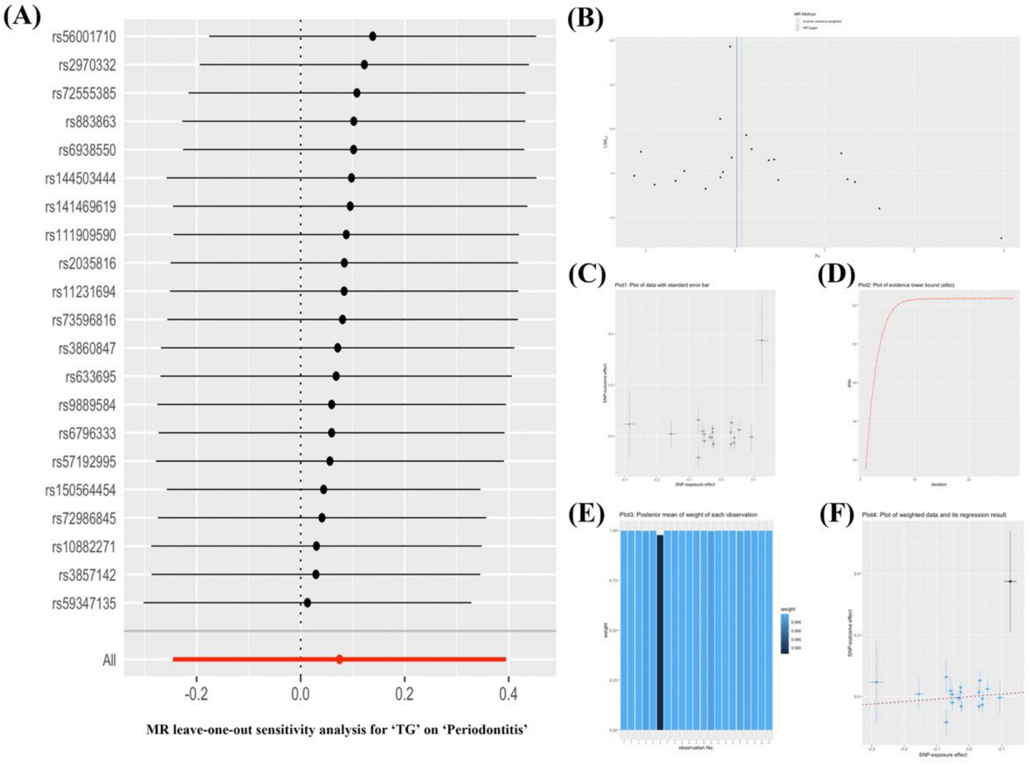

Supplementary Figure 10. Complementary MR analyses of TG with periodontitis of the validation cohort.

A. Leave-one-out sensitivity analysis, removed each SNP in turn and then performed MR repeatedly to eliminate the influence of a single SNP; B. Funnel plot, tested for potential asymmetry; C. Plot of data with standard error bar in BWMR. Dots represent the estimated causal effect sizes (Beta), and their standard errors (SE) are represented by bars; D. Plot of evidence lower in BWMR; E. Posterior means of the weight of each observation in BWMR, valid SNPs were assigned close to 1 and outliers were adaptively down-weighted by BWMR; F. Plot of weighted data and its regression result in BWMR. The dots represent the causal effect (Beta) for each SNP; the bars represent their standard errors (SE); the dashed line indicates the regression slope for BWMR, and the depth of blue means the weight.
